# Methylmercury Effect and Distribution in Two Extremophile Microalgae Strains *Dunaliella salina* and *Coccomyxa onubensis* from Andalusia (Spain)

**DOI:** 10.3390/microorganisms12030434

**Published:** 2024-02-21

**Authors:** Samuel Simansky, Jiří Holub, Ivana Márová, María Cuaresma, Ines Garbayo, Rafael Torronteras, Carlos Vílchez, Zivan Gojkovic

**Affiliations:** 1 Algal Biotechnology Group, Centro de Investigación y Desarrollo de Recursos y Tecnologías Agroalimentarias (CIDERTA), University of Huelva, 21007 Huelva, Spain; samuel.simansky@vut.cz (S.S.); maria.cuaresma@dqcm.uhu.es (M.C.); garbayo@dqcm.uhu.es (I.G.); bital.uhu@gmail.com (C.V.); 2 Faculty of Chemistry, Brno University of Technology, Purkyñova 118, 61200 Brno, Czech Republic; jiri.holub2@vut.cz (J.H.); marova@fch.vut.cz (I.M.); 3 Department of Environmental Biology and Public Health, Experimental Science Faculty, University of Huelva, 21007 Huelva, Spain; torronte@dcaf.uhu.es

**Keywords:** methylmercury, microalgae, mercury contamination, mercury toxicity

## Abstract

The main entrance point of highly toxic organic Hg forms, including methylmercury (MeHg), into the aquatic food web is phytoplankton, which is greatly represented by various natural microalgal species. Processes associated with MeHg fate in microalgae cells such as uptake, effects on cells and toxicity, Hg biotransformation, and intracellular stability are detrimental to the process of further biomagnification and, as a consequence, have great importance for human health. The study of MeHg uptake and distribution in cultures of marine halophile *Dunaliella salina* and freshwater acidophilic alga *Coccomyxa onubensis* demonstrated that most of the MeHg is imported inside the cell, while cell surface adhesion is insignificant. Almost all MeHg is removed from the culture medium after 72 h. Significant processes in rapid MeHg removal from liquid medium are its abiotic photodegradation and volatilization associated with algal enzymatic activity. The maximum intracellular accumulation for both species was in 80 nM MeHg-exposed cultures after 24 h of exposure for *D. salina* (from 27 to 34 µg/g_DW_) and at 48 h for *C. onubensis* (up to 138 µg/g_DW_). The different Hg intakes in these two strains could be explained by the lack of a rigid cell wall in *D. salina* and the higher chemical ability of MeHg to pass through complex cell wall structures in *C. onubensis*. Electron microscopy studies on the ultrastructure of both strains demonstrated obvious microvacuolization in the form of many very small vacuoles and partial cell membrane disruption in 80 nM MeHg-exposed cultures. Results further showed that *Coccomyxa onubensis* is a good candidate for MeHg-contaminated water reclamation due to its great robustness at nanomolar concentrations of MeHg coupled with its very high intake and almost complete Hg removal from liquid medium at the MeHg levels tested.

## 1. Introduction

Mercury (Hg) is an omnipresent trace metal widely distributed in the natural environment. It is highly toxic to living organisms and prolonged exposure can cause severe central nervous system impairment, respiratory insufficiency, and potentially lethal consequences [1]. Specific studies have also recorded developmental setbacks in children, along with unfavorable impacts on cardiovascular health and the immune system [2]. Among the Hg species, the organic methylated variant of Hg, known as methylmercury or MeHg, is one of the most pernicious contaminants [3]. MeHg is also a very toxic organic Hg form that accumulates in the brain and liver with similar toxic effects to inorganic Hg with the addition of reproductive impairment [4]. MeHg owes its high toxicity to its pronounced binding affinity to proteins, which also facilitates its accumulation within tissues. Consequently, this induces biomagnification throughout the entire aquatic food web, spanning from phytoplankton to upper-level predators [5,6].

During the past century, human-induced discharges of Hg including the combustion of fossil fuels, the manufacturing of non-ferrous metals, iron and steel production, waste incineration, cement production, and several other industrial operations have escalated the atmospheric levels of Hg by at least a factor of three [7,8]. Moreover, the expansion of seafloor sediments, which serve as an optimal habitat for mercury-methylating and sulfate-reducing bacteria, is perpetually amplifying due to the anthropogenic eutrophication of aquatic systems and global warming [9]. 

The primary pathway for Hg entry into the food chain is the uptake and bioconcentration by phytoplankton [10]. The levels of MeHg in phytoplankton and zooplankton can be approximately 10^5^ to 10^6^ times greater than the concentration of MeHg in seawater [11]. 

Thus, exposure of phytoplankton even to low concentrations of inorganic Hg (IHg) or MeHg can significantly endanger the functionality of entire aquatic systems and consequently pose a threat to human health through the consumption of seafood [12,13].

Evidence suggests that exposure to high concentrations of both Ihg and MeHg causes toxic effects in primary producers, including a reduced growth rate, a decrease in photosynthetic activity, and extremely high oxidative stress [12,14,15]. Fortunately, the concentrations of Hg typically present in water remain well below the thresholds that significantly impact the photosynthesis and growth of microalgae [16]. The initial defense strategy of microalgae is to alleviate the presence of toxic Hg particles by reducing the reactive cell surface area with fewer ligands, thereby limiting the absorption of the metal [15,17]. Another strategy is the immobilization of Hg on the cell surface, which can effectively mitigate its toxic effects. Certain sources propose that as much as 56% of the total accumulated cellular Hg can be stored within the cellular debris fraction [18]. The third strategy involves the utilization of intracellular sulfur-rich complexes to sequester the present Hg, thereby regulating its intracellular speciation and facilitating its compartmentalization into vacuoles [15,17,19]. This study’s aim was to assess the methylmercury exposure, with subsequent Hg bioaccumulation and distribution, in liquid cultures of two extremophilic microalgal strains: marine alga *Dunaliella salina* and freshwater *Coccomyxa onubensis*, native to Andalusia, Spain. Extremophiles have been shown to display unique, efficient mechanisms to remove metal species from contaminated water. This is the first study, to the best of our knowledge, dealing with MeHg interaction with *Coccomyxa onubensis*. The obtained results suggest that both strains have the potential to be used as bio-agents with the ability to remove and bioaccumulate MeHg from contaminated marine and freshwater aquatic environments.

## 2. Materials and Methods

### 2.1. Microalgal Strains Used in the Study and Standard Cultivation Conditions

Two microalgal strains were used in this study: the freshwater acidophilic microalga *Coccomyxa onubensis* ACVV1—locally isolated from the Rio Tinto River, Huelva (Spain) [20]—and the marine halophile *Dunaliella salina* CCAP 19/30 acquired from the strain collection of the Institute of Plant Biochemistry and Photosynthesis of the CSIC (La Cartuja, Seville, Spain). 

The microalga *C. onubensis* was cultivated in K9 aqueous medium [21], while *D. salina* was cultivated in Gulliard’s (F/2) seawater culture medium [22]. Both culture media were prepared without the addition of Zn^2+^ and Cu^2+^ salts and Na_2_EDTA to prevent their interference with MeHg in the process of internalization, as well as EDTA-mediated MeHg stabilization or chelation in the aqueous medium, as explained in [23].

All glassware used in the study was previously washed with a 50% HNO_3_ Suprapur^®^ solution for 24 h to eliminate any type of impurity contained in said materials and avoid interferences in the results. 

During the experiments, samples were taken daily from each culture in order to perform different measurements: pH, optical density (OD at λ = 530, 680, and 750 nm), biomass dry weight, and quantum yield (Qy) (using an Aquapen PAM fluorimeter AP100, Photon Systems Instruments, Brno, Czech Republic). Samples were also observed under an optical microscope to confirm the absence of biological contamination of any kind.

### 2.2. Measurements of the Optical Density (OD), Biomass Concentration (g_DW_/L), and Quantum Yield (Qy) of the Cultures

The optical density (OD) of the culture was measured at three wavelengths (530, 680, and 750 nm) using a UV/Visible spectrophotometer (Evolution 201, Thermo Fisher Scientific Waltham, MA, USA) in a 10 mm light path polystyrene cuvette. The biomass concentration was measured as dry weight to calculate Hg distribution in all measured fractions—and adhered to the plastic surface—after 72 h of cultivation. In order to express biomass concentration at the end of the experiments for each replicate and assess the MeHg concentration, a calibration curve of optical density at 750 nm versus biomass concentration (DW) was constructed for each algal strain. For the microalga *C. onubensis,* the calibration equation was

(1) $$DW(g/L)=0.2841\times OD\left(750\right)-0.0173\;\mathrm{with}\;R^{2}\;=\;0.9964;$$

For the microalga *D. salina*, the calibration equation was

(2) $$DW(g/L)=1.3474\times OD\left(750\right)+0.1435\;\mathrm{with}\;R^{2}\;=\;0.9978;$$

The biomass concentration (in g/L) was determined as described in [24] after filtration of a known culture volume over pre-dried and pre-weighed glass fiber filters (FMV5, Filtros Anoia, Barcelona, Spain) by measuring the weight increase in the dried filters. The biomass on filters of *D. salina* was washed twice with 150 mL of ammonium formate (NH_4_HCO_2_) isosmotic to the culture medium to remove excess salts [25]. The biomass on filters of *C. onubensis* was washed twice with 150 mL of demi water to remove excess salts. Finally, the biomass concentration was calculated and expressed in g/L of the culture [24]. 

Photosystem II (PSII) maximum quantum efficiency (Qy) was determined by measuring the chlorophyll fluorescence in a portable pulse amplitude-modulation (PAM) fluorimeter (AquaPen AP-100, Photon Systems Instruments, Brno, Czech Republic) according to [26]. 

### 2.3. Experimental Setup and Sample Preparation

Inoculation was performed by adding 5% *v*/*v* of fresh inoculum (OD 750 nm ≥ 1.0) to the total volume of the new culture medium. Cultures were diluted to an initial optical density of OD (750 nm) = 0.5 ± 0.1 [23,27]. Afterward, the MeHg exposure experiments were carried out. For each species, three MeHg exposure concentrations (15, 30, 80 nM) were tested. This study was divided into three consecutive phases. In the first phase, microalgae were exposed to MeHg in Sarstedt cell culture flasks T-175 made of polystyrene (PS) with a 125 mL working volume (500 mL of total volume) and an illuminated surface of 175 cm^2^ (Sarstedt, Sarstedt, Germany). Two replicates were cultivated for each species and for each MeHg concentration, obtaining four total replicates per exposure level. The corresponding concentration of MeHg was added to each bottle and the flasks were placed on an orbital shaker (PSU 20i Orbital Shaker—Bonsai Lab, Alcobendas (Madrid) Spain) rotating at 115 rpm and illuminated with an LED panel providing 650 µmol/m^2^/s of PAR intensity with a photoperiod of 12 h light and 12 h dark [23].

The second phase was the screening of culture growth with the addition of 2 different stressors (80 nM MeHg and increased NaCl concentrations in culture medium: 150 and 300 mM NaCl for *C. onubensis* and 1.0 and 1.4 M NaCl for *D. salina*). We tested the effect of various levels of MeHg and NaCl on algal culture growth in a 6-well plate (6 × 5 mL—under the same conditions of shaking and light exposure as the 125 mL cultures from the previous phase), using the Multiscan FC, (Thermo Fisher Scientific Waltham, MA, USA) plate reader. Based on these screening results, we proceeded to the third phase.

The third phase was performed using 1.8 L bubbled cultures exposed to both NaCl (salt) stress and 80 nM of MeHg. Each 2.0 L Duran bottle was illuminated with a flexible LED panel of 350 µmol/m^2^/s that covered the bottle completely and provided intensive white LED light (in 8 h light/16 h dark cycles). The cultures were monitored over 72 h, with sampling at 0, 24, 48, and 72 h, and OD, pH, Qy, and Hg levels were determined at these sampling points. To achieve the three different concentrations of MeHg used in this study (15, 30, and 80 nM of MeHg), 1.0 g/L commercial stock was used (standard certified solution 1000 ppm MeHg chloride, Sigma Aldrich, St. Louis, MA, USA). 

### 2.4. Intracellular Microstructure Examination of the Microalgal Cells by Transmission Electron Microscopy (TEM)

For observations in TEM microscopy, cells exposed to the highest MeHg concentration (80 nM), as well as untreated cells (control) after 72 h of culturing as explained above, were used. Cells were prepared and observation by TEM was performed as explained in [24]. Briefly, algal cells were collected from each culture and fixed with 1% glutaraldehyde in 0.1 M sodium cacodylate buffer (pH = 7.4) for 2 h at 4 °C. The cells were then washed three times using the same buffer. The samples were post-fixed with 1% osmium tetroxide in 0.2 M cacodylate buffer at 4 °C for 1 h. Samples were washed with the same buffer, dehydrated in a graded ethanol series, and embedded in Epon 812 (EMbed 812 Kit; Electron Microscopy Science, Hatfield, PA, USA). Ultrathin sections of 80–90 nm, obtained by an ultramicrotome (UCT, Leica, Wetzlar, Germany) and placed on nickel grids, were stained with aqueous 1% (*w*/*v*) uranyl acetate and lead citrate. Transmission electron micrographs were observed with a JEM 1011 (JEOL Ltd., Tokyo, Japan) electron microscope using an accelerating voltage of 80 kV. Several photographs of entire cells and of local detailed structures were randomly taken, analyzed, and compared to investigate MeHg’s effect on the different subcellular structures of both species used in this study. 

### 2.5. Superoxide Dismutase Activity

Superoxide dismutase (SOD) activity was based on the [28] method, as described by [29]. The method quantifies the inhibition of nitroblue-tetrazolium (NBT) staining (at λ = 530 nm) due to the decrease in SOD. Each unit of SOD was defined as the amount of enzyme required to inhibit 50% of the reaction of superoxide anions with NBT. 

Protein content was determined spectrophotometrically in the crude extract using the Lowry method [30], by interpolation on a bovine serum albumin (BSA) standard curve, measured at 580 nm [29]. Crude extracts prepared for SOD activity were diluted with SOD extraction buffer. More details of the SOD reagent can be found in [29]. The protein content of the samples was determined spectrophotometrically with absorbance measurement at 580 nm, using distilled water as a blank. 

### 2.6. Sample Preparation for Mercury Determination via Atomic Absorption Spectroscopy (AAS) Technique

For each species, three MeHg exposure concentrations (15, 30, and 80 nM) and two additional controls were carried out: biotic control of algae without MeHg, as well as the abiotic control of culture medium with 80 nM MeHg. The biotic control was used to determine the growth rates of microalgae without MeHg, and it did not contain any detectable amount of Hg. The abiotic control was used to measure the photodegradation of MeHg in the time course of the experiment and its eventual adhesion to the plastic flask surface. At the end of the abiotic control experiments, flasks were emptied and dried, then filled with 6 mL of 20 nM Na_2_EDTA solution and left on the shaker for another 8 h. The AAS results were interpreted as the mercury absorption capacity of polystyrene surface for the cell culture T-175 bottles used for culturing algae at a 125 mL volume (first phase). All exposure experiments in the 125 mL Sarstedt culture flasks lasted for 72 h with four sampling times: 0, 24, 48, and 72 h. At each sampling time, 35 mL of culture volume was taken under sterile conditions inside the laminar flow cabinet. The sample was centrifuged at 3000× *g* (Eppendorf 5702 centrifuge, Hamburg, Germany) for 10 min and the supernatant was separated from the pellet. The pellet was washed three times with 2 mL of 20 nM Na_2_EDTA solution and these aliquots were pulled together. The pellet was freeze-dried using Telstar Cryodos-80 and kept for Hg analysis. Both supernatant and washing solution were immediately acidified with 2% of concentrated HCl and 2% of concentrated HNO_3_ to stabilize Hg present in the samples and analyzed afterward by AAS. 

The determination of Hg in aqueous, liquid, and solid samples via AAS was carried out using the Milestone DMA80 Mercury Analyzer, according to the manufacturer’s instructions. Liquid Hg standards of 80 and 800 ppb were used alongside a TORT-3 clam hepatopancreas reference material, with a certified value of Hg of 0.292 µg/g. To check the reproducibility of the results in our matrix, duplicates of the analyses were performed automatically with 10% of the original sample. The quality of the obtained data was assured by performing technical replicates from duplicates and aliquots of the same sample, which were analyzed separately in the Hg analyzer, and which provided differences between replicates of less than 12%.

### 2.7. Statistical Treatment of the Data 

Statistical analysis was performed using Past4 software v 4.14 (Paleontological Statistics Software Package for Education and Data Analysis) [31]. Statistical differences between different treatments and culture parameters of different samples were tested as follows: normality of the data was tested using Shapiro–Wilk and Anderson–Darling tests; homogeneity of variance was tested by Levene’s test; comparisons between groups were performed using (1) an ANOVA test followed by Tukey’s pairwise test for normally distributed data, or (2) the Kruskal–Wallis test for equal medians followed by the Mann–Whitney unpaired test when a normal distribution of the data could not be assumed. The significance level was set to 0.05.

## 3. Results and Discussion

### 3.1. Growth Performance of Microalgae Exposed to MeHg 

**Figure 1:**
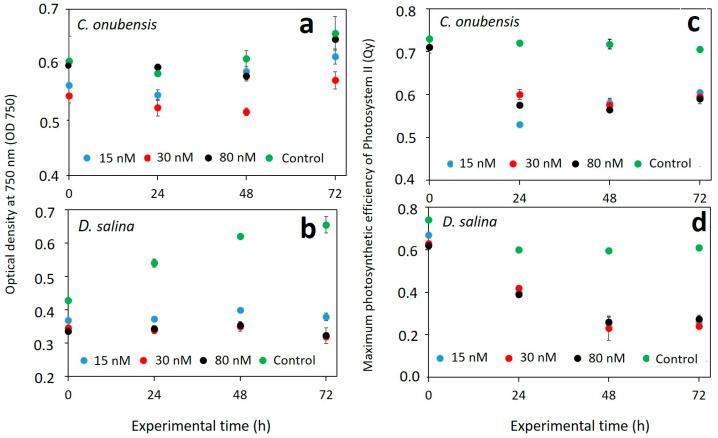
Left: Optical density measured at λ = 750 nm in the function of the exposure time (72 h) for microalga *C. onubensis* (**a**) and microalga *D. salina* (**b**) cultures with 15, 30, and 80 nM of added MeHg as well as the control culture (no MeHg). Right: Maximal photosynthetic efficiency of Photosystem II measured as quantum yield (Qy) in the function of the exposure time (72 h) for microalga *C. onubensis* (**c**) and microalga *D. salina* (**d**) cultures with 15, 30, and 80 nM of added MeHg as well as the control culture (no MeHg). Error bars present standard deviation values of four measurements from two biological replicates with two technical replicates for each measuring point.

Optical density measured at λ = 750 nm is often used as an indicator for the nonspecific microalgal biomass growth as it is not related to any pigment content [23,26]. Optical density (OD 750) measurements that were used as the indicator of *C. onubensis* culture growth are presented in Figure 1a. All cultures exhibited an initial decline with overall moderate growth at the end of the experiment (72 h). There was a statistical difference among cultures exposed to 15 and 30 nM of MeHg and control culture as these were far more affected by MeHg addition. Lower values of the OD in *C. onubensis* cultures exposed to 30 nM MeHg (Figure 1a) compared to 80 nM exposed cultures can be explained by the lower initial optical density of the cultures upon inoculation. Nevertheless, all MeHg-exposed cultures increased their OD in 72 h of cultivation compared to the inoculum despite obviously being affected by MeHg to a certain degree. This demonstrates that MeHg in the applied range was not lethal to algae and could be tolerated by the different cell mechanisms. According to Figure 1c, Qy of MeHg-exposed cultures decreased compared to control but with no obvious Qy differences among MeHg-exposed cultures. Interestingly, *C. onubensis* cultures exposed to 80 nM MeHg had an optical density only slightly lower than control (Figure 1a) while their Qy was affected by MeHg and decreased from 0.7 to 0.6 during 72 h of experiments. These differences in culture growth and photosynthetic efficiency decrease upon MeHg exposure can be explained by the specific mode of action of MeHg on the cell and certain limitations of the culture growth in shaken cultures of 125 mL.

Optical density (OD 750) measurements were used as indicators of *D. salina* culture growth and are presented in Figure 1b. It is obvious that *D. salina* was more affected by MeHg in the tested concentration range. In this case, there was a statistical difference between the control culture and all exposed cultures. While exposed cultures had a very moderate (almost flat) OD change over time, the control culture increased its optical density from 0.4 to 0.63 in 72 h of the experiment. It was already reported that when treated with different non-lethal concentrations of MeHg, microalgae tend to grow similarly among treatments with no correlation between final algal densities and bioconcentration of MeHg in the cell biomass [32]. 

### 3.2. Effect of MeHg on the Culture Health and Photosynthetic Activity

The maximal photosynthetic efficiency of Photosystem II is widely recognized as a fast, reliable, and effective method to assess algal photosynthetic performance and overall culture health [33]. The maximal photosynthetic efficiency of Photosystem II was measured using the PAM amplitude modulation technique and expressed as quantum yield (Qy), which is a unitless and positive number less than 0.9. The Qy evolution in the 72 h of the experimental time for both species is shown in Figure 1c,d. Qy values above 0.6–0.7 are considered normal values of healthy cultures, while lesser values suggest stressed cultures with partially impaired photosynthesis [20,24,33]. From Figure 1, it can be concluded that all the *C. onubensis* cultures had viability in the 72 h of experiments (Figure 1c), while *D. salina* was strongly affected (Figure 1d). In both strains, there was a significant difference (*p* ≤ 0.02) between control cultures and cultures exposed to 15, 30, and 80 nM of MeHg. However, there was no significant difference among MeHg-exposed cultures in both species, suggesting that the selected MeHg range was non-lethal to these strains.

In *C. onubensis* cultures exposed to MeHg, initial Qy values decreased during the first 24 h, and from that time point on, it gradually increased to healthy culture values (Qy = 0.6) towards the end of the experiments, suggesting culture adaptation to the MeHg addition. But as previously mentioned, in *D. salina* cultures, the photosynthetic efficiency was more affected compared to *C. onubensis* as Qy decreased significantly at 72 h of exposure. It is well known that when Hg enters the algal cell, it affects various physiological processes, including photosynthesis [34]. It was also reported that even 10–100 pM of MeHg had a hormetic effect on the photosynthetic efficiency of the green alga *C. reinhardtii* [35]. In the blue–green algae *Spirulina platensis,* exposure to 20 µM Hg for 2 h also induced a decrease in the quantum yield and partial changes in Photosystem II photochemistry [36]. Exposure of the alga *T. pseudonana* to 8 nM MeHg for 72 h significantly decreased both the Qy and operational quantum yield (Φ_M_) of the culture [37].

Superoxide dismutase (SOD) activity was measured for both algae at the highest MeHg concentration (80 nM) and compared to the SOD activity of the corresponding control cultures. When exposed to high concentrations of metals, microalgae increase the activity of different antioxidant systems and their enzymatic response mechanisms, including SOD, catalase, glutathione peroxidase, glutathione reductase, and ascorbate peroxidase, and activate the synthesis of low-molecular-weight compounds such as glutathione and carotenoids [10,29]. Enzyme SOD activity is readily used as an indicator of oxidative stress in algal cells. In *C. onubensis,* specific SOD activity increased from 100 to 160 units/mg of proteins when 2 mM of Fe(III) was added to the culture and cultivated for 11 days [29]. Our results show that there were no significant differences between SOD activity in MeHg exposed and control cultures as the addition of the highest concentration of 80 nM MeHg did not induce any increase in SOD activity upon 72 h of exposure (Table 1). It was reported that a notable SOD activity increase with increased exposure concentration of metal cations is a sign of a more active antioxidant response of the microalga [29]. Our results indicate that the MeHg concentration in the range used in this study (15–80 nM) does not induce an SOD activity increase nor a consequent cellular ROS increase during the first 3 days of cultivation, which suggests that the MeHg mode of action and its toxicity target alternative mechanisms in algae to efficiently cope with the organic Hg species. For these reasons, we proceeded to TEM analysis of the biomass to try to reveal any physical damage or thylakoid alterations in the cell ultrastructure that could be attributed to the MeHg exposure. 

Exposure of *C. reinhardtii* to 10 pM–10 nM of MeHg had a very limited effect on cellular ROS and oxidative stress, suggesting that the microalgae could efficiently cope with the range of tested concentrations for a short period of time [38]. The authors also reported that MeHg-induced oxidative stress enters at much higher concentrations than those used in this study (15, 30, and 80 nM of MeHg). At rather high MeHg concentrations of 0.1 M, the microalga *C. reinhardtii* shows a strong anti-oxidant response including the expression of genes involved in metal chelation and coding for the enzymes catalase and SOD [38]. One proposed mechanism of intracellular Hg detoxification is its binding to thiols containing sulfhydryl groups like glutathione and phytochelatins that strongly bind to Hg inside the cell, rendering it harmless [34,39]. 

### 3.3. Culture Growth Performance in Up-Scaled Cultures (V = 1.8 L) Exposed to 80 nM MeHg and Addition of Higher Levels of NaCl (Salt Stress)

**Figure A1:**
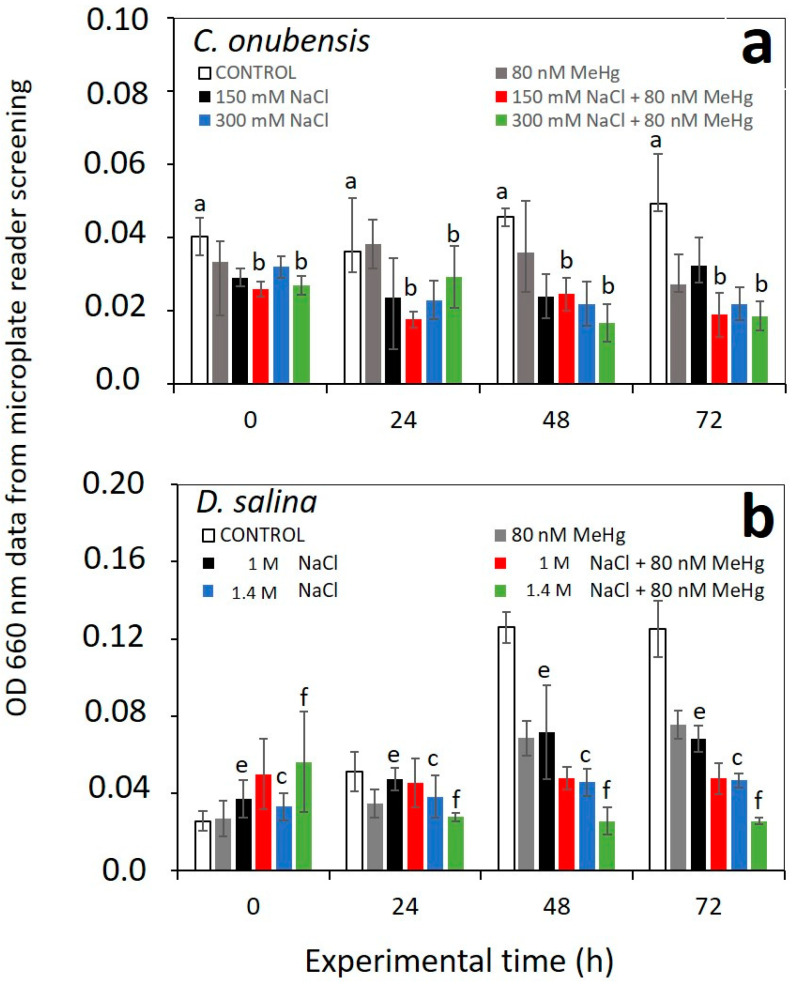
Screening results obtained using microplate reader (6-well plate of 6 × 5 mL) at optical density (OD at λ = 660 nm) in the function of experimental time (72 h) for (**a**) *C. onubensis* exposed to 80 nM MeHg, 150 nM of NaCl, 300 mM of NaCl, or the combination of each salt concentration with addition of 80 nM MeHg. Small letters represent statistical differences: a—different from all other treatments, b—different from culture exposed to 80 nM MeHg; (**b**) *D. salina* exposed to 80 nM MeHg, 1 M of NaCl, 1.4 M of NaCl, or the combination of each salt concentration with addition of 80 nM MeHg. Small letters represent statistical differences: c—different from culture exposed to 1 M NaCl, e—different from culture exposed to 1.4 M NaCl, f—different from all other treatments. Control cultures consisted of the corresponding culture medium with microalgae cultivated under normal conditions.

The screening results obtained from the microplate reader for the combined resistance of *C. onubensis* and *D. salina* to 80 nM MeHg and NaCl (salt stress) are presented in Figure A1 (Appendix A). The cellular response of microalgae to salt stress has some similarity to MeHg exposure at the gene expression level. Namely, in the microalga *C. reinhardtii,* genes involved in cell wall metabolism and the biosynthesis of lignin glycoproteins and expansin proteins were only up-regulated at a 10 nM MeHg concentration [3]. But salt stress had a similar effect on expansin proteins, which were strongly up-regulated and induced the formation of palmelloids in *C. reinhardtii* [3]. This is an indication that both MeHg and NaCl might have similar modes of action by overproducing biomolecules involved in the extension of cell walls during cell division to cope with and tolerate stress [3]. According to those results, the goal of the screening test (phase 2) was to assess if selected extremophilic algal strains could cope with simultaneous exposure to 80 nM MeHg and elevated salt concentrations. For this, different salt concentrations were used: 150 and 300 mM NaCl for the freshwater species *C. onubensis,* and 1.0 and 1.4 M NaCl for the marine species *D. salina* (1.0 and 1.4 M NaCl). 

From the literature, it is known that the microalga *C. onubensis* can readily grow at 500 mM of NaCl when a gradual adaptation is carried out, although salt exposure induces an increase in vacuole number, a change from an ellipsoid to a spherical cell shape, and an increase in the cell size [20]. The authors suggested a strong adaptation mechanism of *C. onubensis* to salt stress, which was explained by the fact that the biomass productivities and photosynthetic performance of salt-added cultures adapted to high salinity and were similar to control cultures without salt [20]. 

The microalga *D. salina* is (besides *D. viridis*) the most-often-found algal species in natural salt lakes and saline ponds [40]. Naturally found *D. salina* can withstand a wide range of NaCl concentrations—from 9.0 to 200 g/L—while it grows optimally at NaCl concentrations of 100–150 g/L NaCl [40]. 

**Figure 2:**
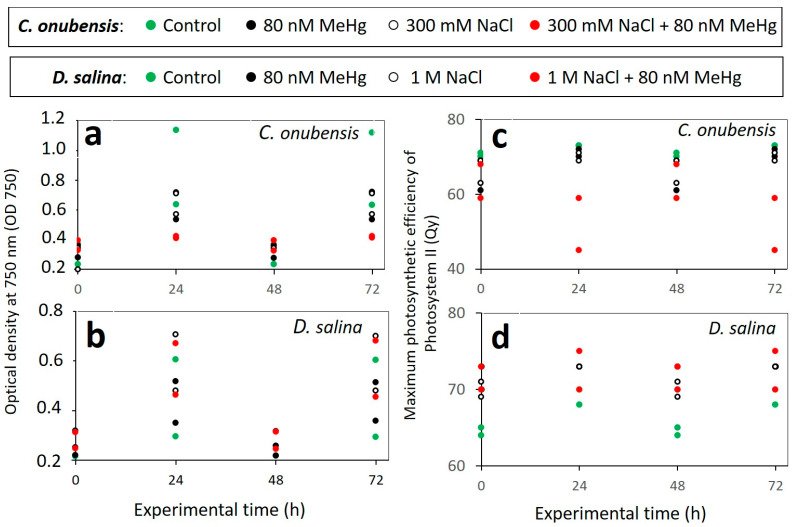
Left: optical density (**a**,**b**) measured at λ = 750 nm in the function of the exposure time (72 h) for microalga *C. onubensis* (**a**) and microalga *D. salina* (**b**) in the 1.8 L bubbled cultures with 80 nM of added MeHg (black circles), cultures with added NaCl (black empty circles), NaCl and 80 nM MeHg (red circles), as well as the control culture (no MeHg, green circles). Right: maximal photosynthetic efficiency (**c**,**d**) of Photosystem II measured as quantum yield (Qy) in the function of the exposure time (72 h) for microalga *C. onubensis* (**c**) and microalga *D. salina* (**d**) with 80 nM of added MeHg (black circles), cultures with added NaCl (black empty circles), NaCl and 80 nM MeHg (red circles), as well as the control cultures (no MeHg, green circles). Salt levels were adjusted for each species: 300 mM NaCl for *C. onubensis* and 1 M NaCl for *D. salina*. There were no statistical differences in OD 750 during the 72 h between control cultures and each treatment for both species. There were statistical differences (*p* < 0.05) in Qy during the 72 h between control culture and each treatment for both species. There were no statistical differences in Qy values in 72 h of exposure among treated cultures.

In this study, both algal species maintained viability during 72 h of combined stress exposure (Figure 2), which initiated further scale-up of these experiments to 1.8 L cultures that allowed us to measure MeHg uptake and distribution in different culture fractions as well as the cell biomass. 

### 3.4. Mercury Distribution and Content in MeHg Exposed Cultures Measured Using AAS

**Figure A2:**
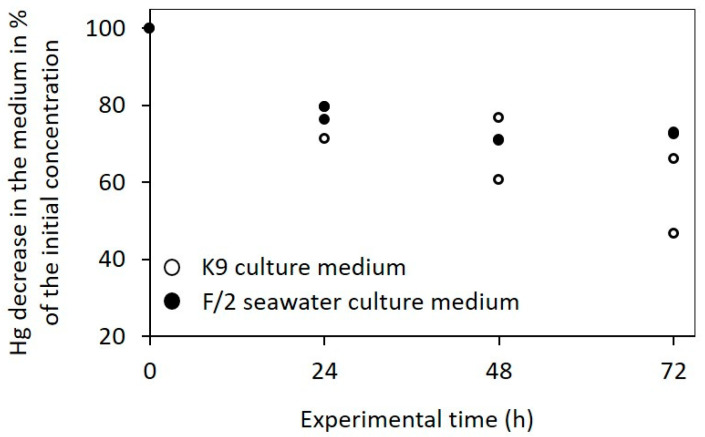
Decrease in the initially added MeHg concentration in the liquid culture medium (measured as Hg) in the abiotic control for K9 culture medium (empty white dots for *Coccomyxa onubensis*) and F/2 seawater medium (black dots for *Dunaliella salina*) in the time course of the experiment (72 h). In the absence of microalgae under the cultivation condition of shaking and illumination in both media, the added MeHg decreased significantly from the initial concentrations. The decrease after 72 h was more pronounced in K9 medium (50%) compared to F/2 seawater medium (30%), which suggests that MeHg is more stable in seawater environments.

The abiotic control demonstrated limited stability of MeHg in aquatic environments without microalgal presence. Figure A2 (Appendix B) presents the decrease in MeHg in the abiotic control of the K9 culture medium (for alga *C. onubensis*) and F/2 seawater medium (for alga *D. salina*) during the time course of the experiment (72 h). In the absence of microalgae under the cultivation condition (shaking and 12 h per day of illumination, as explained in the Materials and Methods section), the MeHg that was initially added decreased significantly from the initial concentrations. This decrease was more pronounced in K9 medium (50%) compared to F/2 seawater medium (30%), which suggests that MeHg is more stable in seawater environments close to neutral pH. It was already observed that light exposure promotes MeHg degradation in algal cultures while prolonged dark periods decrease MeHg uptake by 37% [27]. Photodemethylation of MeHg is a very important process of MeHg degradation in natural surface waters [41]. In natural environments, humic content acts as a natural photoprotector of MeHg by increasing light attenuation and microbial activity [42,43]. It was reported that 24 h of light exposure led to the removal of one-third of the MeHg from the microalgae cultures mainly due to the abiotic loss—including photodegradation that is not related to microalgae directly [44].

**Figure A3:**
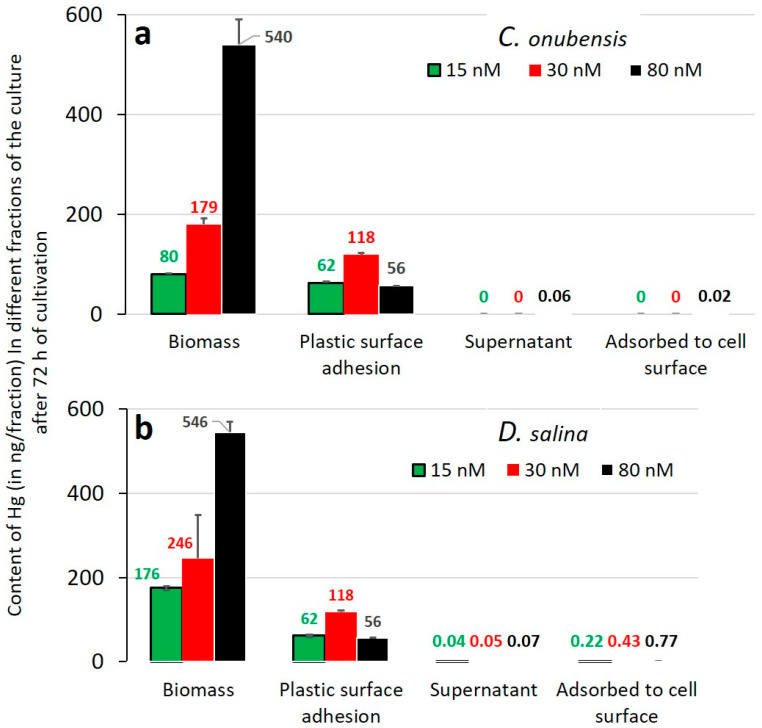
Mercury distribution among different fractions of the 125 mL cultures and culturing bottle polystyrene surface after 72 h of exposure. It was expressed as Hg content (in ng) per fraction: cell biomass, adhesion to plastic surface, supernatant, and cell surface adhesion (determined by Na_2_EDTA cellular washing): (**a**) Hg distribution after 72 h for *C. onubensis*: (**b**) Hg distribution after 72 h for *D. salina*: Green squares represent cultures with 15 nM of MeHg added; Red squares represent cultures with 30 nM of MeHg added, while black squares represent cultures with 80 nM of MeHg added. Small numbers above each bar represent exact value of Hg in that fraction in ng. Number colors correspond to three concentrations of MeHg (15, 30, and 80 nM).

Our results also showed that the plastic (polystyrene) surface of the culture flasks, which was in direct contact with the culture medium added with MeHg, also adhered to some portion of the initially added MeHg. After 72 h of exposure (cultivation) in 15 nM and 30 nM MeHg-added cultures, the plastic surface adhered to 16.1–17.1% and 15.3–16.1% of the initially added MeHg concentration, respectively. In the case of the highest MeHg concentration (80 nM of MeHg), the plastic surface on the bottom of the culture bottle adhered the 2.80–2.83% of the initial MeHg added, which suggests that this plastic material (polystyrene) has a stable, passive Hg-adhering capacity that is not related to the MeHg concentration in the medium. Data on Hg adhesion to the plastic surface of the culture bottles were also included in the MeHg balance and distribution at the end of experiments in 125 mL flasks (Figure A3, Appendix B).

**Figure 3:**
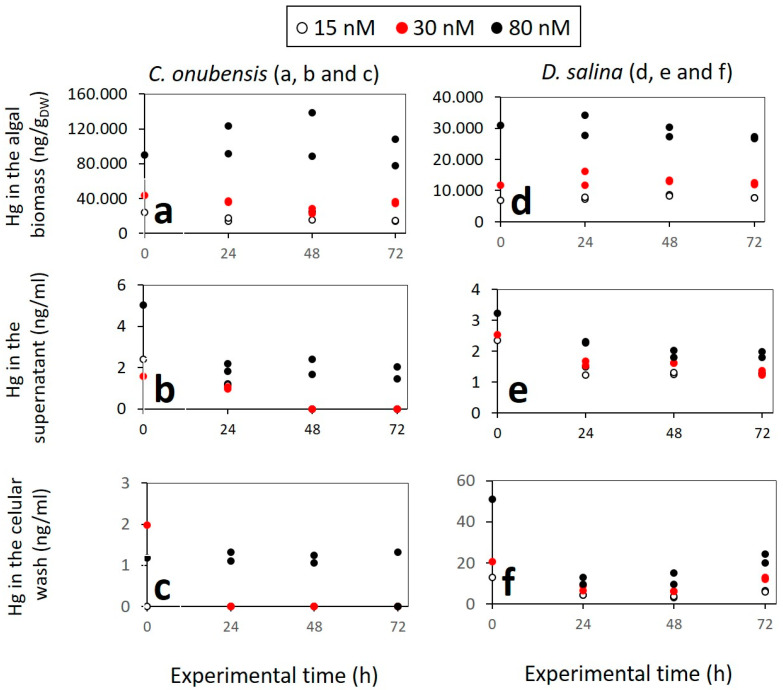
Mercury distribution and content measured by AAS in 125 mL cultures of (**a**) *C. onubensis* biomass (in ng/g_DW_), (**b**) supernatant (in ng/mL), and (**c**) cellular wash with EDTA (in ng/mL); of (**d**) *D. salina* biomass (in ng/g_DW_), (**e**) supernatant (in ng/mL), and (**f**) cellular wash with Na_2_EDTA—which represents Hg adhesion to the cell surface. White circles represent cultures added with 15 nM of MeHg; Red dots represent cultures with 30 nM of MeHg added, while black dots represent cultures with 80 nM of MeHg added.

**Figure 4:**
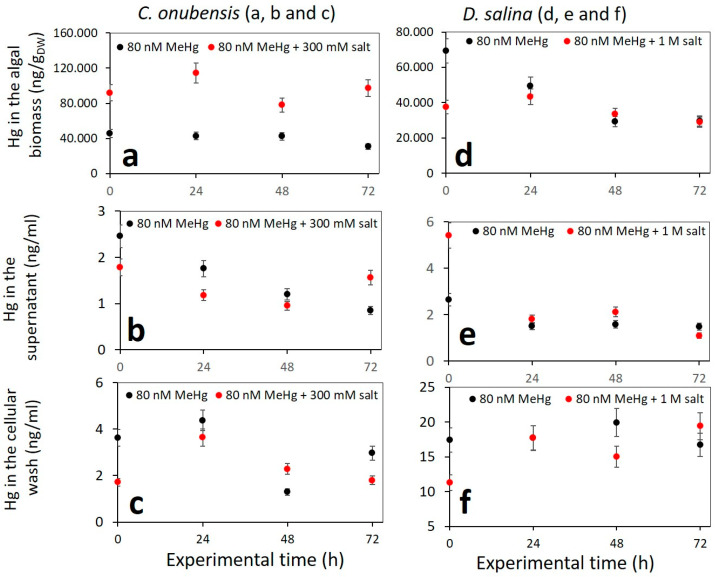
Mercury distribution and content in the scaled-up (1.8 L) cultures of the two selected strains exposed to 80 nM MeHg and elevated concentrations of NaCl (salt stress); for *C. onubensis*: (**a**) algal biomass (in ng/g_DW_), (**b**) supernatant (in ng/mL), and (**c**) cellular wash with EDTA (in ng/mL); for *D. salina*: (**d**) algal biomass (in ng/g_DW_), (**e**) supernatant (in ng/mL), and (**f**) cellular wash with EDTA obtained by AAS. Black dots represent cultures of both species added with 80 nM of MeHg. Red dots represent cultures with 80 nM of MeHg and 300 mM of NaCl added for *C. onubensis* or cultures with 80 nM of MeHg and 1.0 M of NaCl added for *D. salina*.

The distribution of MeHg and its content (measured as Hg) in the culture fractions (intracellular content of the biomass, supernatant, adhesion to the cell surface, and adhesion to plastic) of *C. onubensis* and *D. salina* in 125 mL cultures is presented in Figure 3. Moreover, the distribution of MeHg and its content in the culture fractions (intracellular content of the biomass, supernatant, and adhesion to the cell surface) of *C. onubensis* and *D. salina* in 1.8 L cultures is presented in Figure 4. 

From these data, we can draw some general conclusions:

- Most of the MeHg is imported inside the algal cell: *C. onubensis* accumulates approx. four times more MeHg than *D. salina* at all tested concentrations (15, 30, and 80 nM MeHg). The amount of intracellular Hg is proportional to the initial MeHg added and decreases greatly from 80 nM to 15 nM cultures. For example, intracellular Hg in *C. onubensis* ranged from 77 to 108 µg/g_DW_ in 80 nM culture and 14 to 15 µg/g_DW_ in 15 nM MeHg culture. And intracellular Hg in *D. salina* ranged from 27 to 34 µg/g_DW_ in 80 nM and 7.6 to 7.7 µg/g_DW_ in 15 nM MeHg culture. The maximum intracellular accumulation at the highest concentration of 80 nM MeHg for both species was not at the end of the experiment (72 h) but earlier—at 24 h—for *D. salina* (from 27 to 34 µg/g_DW_) and at 48 h for *C. onubensis* (up to 138 µg/g_DW_)—then it partially decreased till the end of the experiment (Figure 3a,b—black dots), which suggests active biotransformation and removal of Hg possibly via transformation to elemental mercury, which is then removed as exudate from the cell [39]. A similar pattern, although less pronounced, can be observed in 30 nM MeHg-exposed cultures (Figure 3—red dots).
- The high internalization of MeHg in *C. onubensis* was almost doubled by additional exposure to 300 mM NaCl and 80 nM MeHg (Figure 4a), which suggests shared Na and Hg import mechanisms and a common cell defense strategy. However, the addition of salt to the 80 nM MeHg culture in *D. salina* had no effect on MeHg accumulation in the 72 h time period (Figure 4b). Salt addition also had no obvious effect on MeHg removal from the supernatant nor adsorption to the cell surface in either of the studied microalgae.
- MeHg content in the supernatant was very low throughout the experiment for both species and both culture volumes, and almost all MeHg was removed from the liquid medium at the end of the experiment (*c*(Hg) ≤ 1.99 ng/mL).
- MeHg that was removed from the cell surface (using 0.1 M Na_2_EDTA solution) contained a very low amount of Hg. Nevertheless, the Hg amount at 80 nM MeHg was ten times higher for *D. salina* (20 to 24.2 ng/mL) compared to *C. onubensis* (≤1.99 ng/mL), which was almost identical to the very low supernatant Hg content.
- The difference in cell surface adhesion and intracellular accumulation of MeHg in the tested species can be attributed to the difference in the cell surface structure of these two species. The microalga *C. onubensis* has a thin but rigid cell wall similar to other *Trebouxiphyceae* strains, while *D. salina* completely lacks a cell wall and has only a thin plasmalemma, enabling it to withstand osmotic pressure in high-salinity environments. The other indication is that MeHg has a greater chemical ability to pass through the cell wall chemical structure of *C. onubensis*, perhaps due to its inclusion as a component of amino acid-based hydrophilic complex carriers, which eases its transit through cell walls [45]. Besides photodegradation, the abiotic loss can be associated with the presence of naturally accompanying bacteria in the culture medium that can induce MeHg demethylation [46].

From Figure A3 (Appendix B), it is obvious that the Hg originating from MeHg added to the microalgal culture—upon 72 h of cultivation—was mainly imported intracellularly in the biomass, and a much smaller part adhered to the culture vessel surface while negligible amounts were still present in the supernatant or adhered to the cell surface. These data underline the importance of two processes: (1) microalgal activity in the MeHg removal by internalization or volatilization, and (2) abiotic and photochemical activity that removes a great part of MeHg even when algae are not present (Figure A2 Appendix B). The uptake of MeHg by phytoplankton is a very important step in its further removal because the biotic transformation of MeHg in algal cultures is considered to be a mainly intracellular process [47]. Previous works showed that the amount of Hg remaining in the growth medium of *Chlorella autotrophica* was very low, suggesting the transformation of Hg cations into the volatile form of elemental Hg^0^ in the presence of some mercury-reducing factors in *Chlorella* cells [37]. The internalization of the picomolar levels of the mixture of isotopically enriched inorganic (^202^Hg and ^201^Hg) and methylmercury (^202^MeHg and ^199^MeHg) in *C. reinhardtii* suggested the existence of oxidative demethylation mechanisms in algae. While the opposite process—methylation of the inorganic Hg inside the cell—was not detected [39]. Another study on the MeHg intake and distribution in the biomass of different algal species demonstrated that the highest percentage of intracellular MeHg was found in the heat-stable protein fraction of the biomass (44%, 68%, and 80% MeHg in *T. pseudonana*, *C. autotrophica,* and *I. galbana*, respectively) while less than 30% of MeHg was associated with the cellular debris and organelle compartments [37]. And the natural consortium of freshwater algae exposed to 10 ng/L of MeHg accumulated up to 300 ng/g_DW_ in the time period of 6 h [44]. As can be inferred from these studies, the accumulation of MeHg in phytoplankton differs among species but generally increases at higher levels of exposure [37]. The same result was obtained in the current study. It was also reported that the amount of intracellular Hg uptake differs greatly among species under the same exposure conditions. For example, the alga *T. pseudonana* accumulates much more intracellular Hg than *I. galbana* [37]. Again, the same trend was found in this study: under the same conditions, *C. onubensis* accumulated approx. four times more intracellular mercury than *D. salina.* The presence of the rigid cell wall in *C. onubensis* represents an additional barrier for Hg uptake compared to *D. salina* [38]. Nevertheless, the higher MeHg accumulation in *C. onubensis* (compared to *D. salina*) suggests predominantly active intake and fast internalization in *C. onubensis*. Differentiation between MeHg that is adsorbed to the cell surface and intracellular MeHg is rather important as it was noticed in *S. capricornutum* culture that rapid MeHg adsorption to the weak cell surface sites may not necessarily lead to MeHg intake and internalization [23]. 

### 3.5. Impact of MeHg on the Ultrastructure of the Microalgal Cells

**Figure 5:**
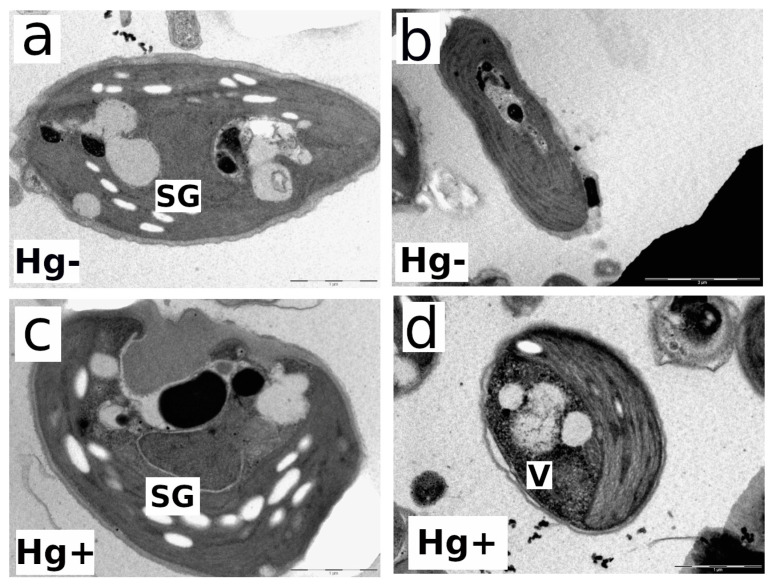
Transmission electron microscopy (TEM) images of the individual cells from microalga *Coccomyxa onubensis*: control culture without MeHg (**a**,**b**); exposed to 80 nM of MeHg for 72 h (**c**,**d**). Abbreviations: (Hg−)—control culture; (Hg+)—culture exposed to 80 nM of MeHg; (SG)—starch granules; (V)—vacuoles. The bar in the bottom-right corner of each image represents 1 µm distance.

Electron microscopy studies on the ultrastructure of both strains were carried out at the end of the experiment (72 h) for the 80 nM MeHg-exposed and control cultures (Hg-free). Figure 5a,b show longitudinal and cross-sections of control cells (MeHg-free culture). *C. onubensis* (a) cells are elongated to an ellipsoidal shape, with approx. 7 µm long and 3 µm wide [20]. The cell has one large chloroplast occupying approx. half of the total cell volume surrounding the nucleus with visible thylakoids and starch bodies. The cell is surrounded by a distinct thin cell wall [20]. Figure 5c,d show an obvious increase in the vacuolization (small gray spots) and disruption and irregularities in the thin cell wall in the MeHg-exposed cells compared to control cells (a,b). MeHg-exposed cells have a visible fingerprint-like thylakoid pattern typical for metal exposure [24] and an increase in electron-dense structures related to Hg internalization. 

**Figure 6:**
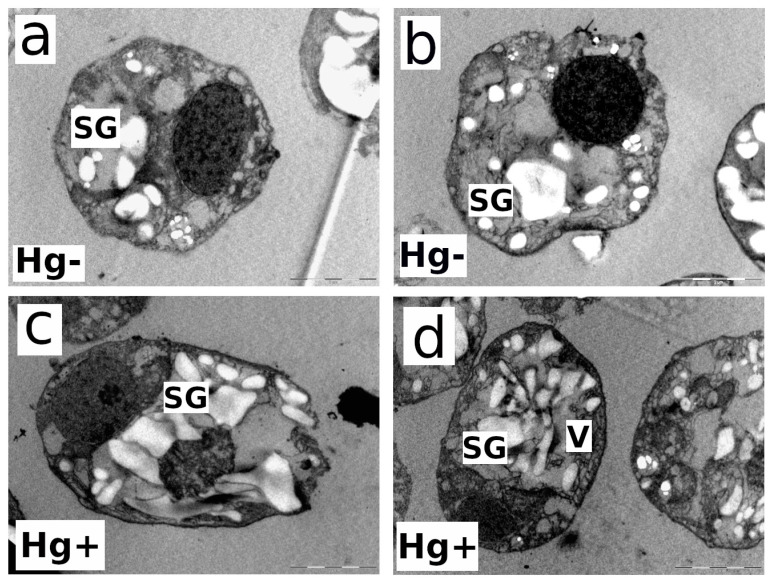
Transmission electron microscopy (TEM) images of the individual cells from microalga *Dunaliella salina*: control culture without MeHg (**a**,**b**); exposed to 80 nM of MeHg for 72 h (**c**,**d**). Abbreviations: (Hg−)—control culture; (Hg+)—culture exposed to 80 nM of MeHg; (SG)—starch granules; (V)—vacuoles. The bar in the bottom-right corner of each image represents 1 µm distance.

*D. salina* has ellipsoidal, ovoid-to-oval solitary cells that vary in size greatly but are usually 16–18 µm long and 10–11 µm wide with two identical flagella at the apical side [48]. The main morphological characteristic of this species is its high plasticity because it lacks a rigid cell wall and has only a thin transparent plasmalemma surrounding the cell. In control cultures, cells have one cup-shaped chloroplast surrounded by starch with a pyrenoid in the center (Figure 3a,b). Oil drops containing mostly β-carotene accumulate in the chloroplast stroma under extreme conditions [48]. The vacuolization process is very well observed in Figure 5d and Figure 6d. In Figure 5d, obvious microvacuolization appears in the form of many very small vacuoles (V). All the small vesicles or vacuoles that are spread throughout the cytoplasm are obvious proof of the increase in vacuolization. In Figure 5c, the largest white granules are starch granules (SG), but there are also gray vacuoles. The starch granules are the most defined, while the vacuoles are less defined and more grayish. In Figure 6d, especially on the right side of the cell, very close to the cell membrane, small vacuoles (not as much as in Figure 5d), grayish in color, are microvacuoles as well. Figure 6c,d show an increase in the vacuolization, as already mentioned above (small gray spots), and cell membrane disruption in 80 nM MeHg-exposed cultures compared to control (MeHg-free) cells (Figure 6a,b). Cells exposed to MeHg also have greater disruption of chloroplast organization and show an increase in electron-dense structures, which is probably related to internalized mercury. As previously commented on, intracellular Hg at higher concentrations binds to physiologically important organelles and chloroplasts, which affects photosynthesis. To cope with internalized Hg toxicity, microalgae use different detoxification mechanisms like the volatilization of Hg and its removal by exudation or its sequestration by physiologically important thiols [34,39]. As discussed in Section 3.4, the obviously different cell surfaces of both strains, the robust, rigid cell wall of *C. onubensis* and the thin plasmalemma of the cell wall-lacking *D. salina* strain, might explain the different cell surface adhesion capacities of the tested species. 

## 4. Conclusions

Both studied strains of microalgae are resistant to MeHg and able to accumulate it when exposed to the nanomolar range (15–80 nM) of MeHg. The highest tested concentration (80 nM MeHg) was not able to induce SOD accumulation, although it had some effect on the culture growth and alterations in the cell’s ultrastructure. The freshwater acidophilic alga *C. onubensis* intracellularly accumulates most of the added MeHg—up to 108 µg/g_DW_ after 72 h of exposure. The marine halophile *D. salina* accumulates less MeHg—up to 27 µg/g_DW_ in the same period of time. After 72 h of exposure, the MeHg content in the liquid culture medium of both species is negligible, and most of it is found imported in the algal cells or eliminated via biological reduction to elemental Hg and/or abiotic, photochemical action not related to algae. The high capacity of both algae to accumulate MeHg from the aquatic environment can be potentially hazardous to zooplankton that feed on microalgae and wildlife located further in the aquatic food web due to the gradual biomagnification of mercury. On the other hand, such a high MeHg accumulation capacity recommends both algae for the bioremediation and water reclamation of MeHg-contaminated seawater and freshwater sources. The internalization of available MeHg in contaminated water could provide fast and easy Hg removal after microalgal biomass separation by flocculation, flotation, filtration, etc. The effectiveness of these two species at a large scale (i.e., in large ponds with mercury-contaminated waters) may differ from these results and merits further investigation.

## Figures and Tables

**Table 1:** Enzymatic SOD activity in the cultures exposed to 80 nM of MeHg for 72 h compared to Hg-free control cultures for microalgae *D. salina* and *C. onubensis*.

Microalga	SOD Activity Units/mL of Cell Extract	SOD Activity Units/mg of Cell Proteins
*C. onubensis* + 80 nM MeHg	16.3–16.5	4.84–7.57
*C. onubensis* (control—MeHg free)	16.3–16.9	5.50–6.48
*D. salina* + 80 nM MeHg	14.5–19.0	2.35–2.95
*D. salina* (control—MeHg free)	15.5–16.3	2.22–2.72

## Data Availability

Data are contained within the article.
